# Supplementary material for: Notch signaling blockade links transcriptome heterogeneity in quiescent neural stem cells with reactivation routes and potential
Source: Sci Adv. 2025 Aug 27;11(35):eadu3189. doi: 10.1126/sciadv.adu3189 (PMC12383255; doi:10.1126/sciadv.adu3189)
Supplement: Supplementary file 1 — Supplementary Text Figs. S1 to S7 Tables S1 to S3 Legends for data S1 to S3 References [file sciadv.adu3189_sm.pdf]

Science Advances
AAAS

# Supplementary Materials for

## Notch signaling blockade links transcriptome heterogeneity in quiescent neural stem cells with reactivation routes and potential

David Morizet *et al.*

Corresponding author: David Morizet, david.morizet@pasteur.fr; Laure Bally-Cuif, laure.bally-cuif@pasteur.fr

*Sci. Adv.* **11**, eadu3189 (2025)
DOI: 10.1126/sciadv.adu3189

**The PDF file includes:**

Supplementary Text
Figs. S1 to S7
Tables S1 to S3
Legends for data S1 to S3
References

**Other Supplementary Material for this manuscript includes the following:**

Data S1 to S3

**Supplementary text : Reclassification of qNSC1 as striatal astrocytes**

We reanalyzed the data from the original mouse study (*12*) with a pipeline developed by the Allen Institute suited to Smart-Seq2 data with few cells, and attempted to assign identities to the resulting clusters. The number of clusters and the classification of oligodendrocytes, neuroblasts and proliferating cells were in good agreement. However, we found that one of the quiescent astroglial clusters expressed high levels of genes associated with astrocytes rather than radial glia-like (RG-like) cells. We also re-analyzed another dataset which collected more cells and reported an identical subdivision of the data (*11*) and obtained the original annotations. Once again, we recovered clusters comparable to the published data but found that the cells initially annotated as qNSC1 had a transcriptional profile similar to that of astrocytes. In particular, they expressed high levels of *Aqp4*, *F3* and *S100b*, which are commonly used to distinguish astrocytes from RG-like cells, as well as *Timp4*, *Cxcl14* and *Slc4a4*, which we recently described as being enriched in astrocytes rather than RG-like cells in mouse (*16*). Several independent studies have now generated scRNA-seq atlases of the SVZ without explicitly reporting the presence of the qNSC1 cluster (*8*, *13*, *15*, *40*, *41*). We re-analyzed all of them to determine whether they contained clusters of cells matching the transcriptional signature of qNSC1 cells and how these cells had been classified. We ultimately excluded the data from (*13*) because their study was tailored to recover a large number of cells at the expense of library complexity and thus risked lacking sensitivity to distinguish between closely related cells. Shah et al. did not report the presence of astrocytes in their datasets but labeled qNSC as aNSC as evidenced by the pattern of expression of *Thbs4* (*41*). The large cluster of cells they labeled as qNSC expressed the same astrocytic genes as those mentioned above, thus their classification into qNSC and aNSC matched the qNSC1 and qNSC2 clusters from (*12*) and (*11*). On the other hand, both (*15*) and (*8*) reported the presence of astrocytes alongside NSCs and our re-analysis confirmed that these astrocyte clusters matched qNSC1 (Fig.S4B). Cebrian-Silla et al. (*8*) profiled a large number of cells with high gene detection per cell and identified several spatially distinct populations of qNSC which were closer to each other than to the astrocyte cluster and we independently reproduced the results from this analysis. Overall, this strongly suggests that the cells that qNSC1 cells are in fact astrocytes. We also recovered data from a whole telencephalon atlas (*44*) and used MetaNeighbor (*43*) to map the astroglial cells from (*11*) to those of the atlas. In this analysis, qNSC1 cells clustered together with telencephalic astrocytes and away from NSCs, supporting their astrocytic identity (Fig.S4C). A latent neurogenic

potential, activated upon stroke, injury or Notch inhibition has been reported in striatal astrocytes (*4*) in particular in the medial striatum, close to the lateral wall of the SVZ. We thus asked if qNSC1 cells might be striatal astrocytes and re-analyzed data from a study on striatal astrocyte (*48*). Our analysis largely agreed with the reported clusters and we found that a subset of cells from the striatum of P90, but not P3 mice bearing a striking similarity to qNSC1 cells. One cluster in particular displayed higher expression of *Crym,* which is also enriched in qNSC1 over qNSC2 and is expressed in astrocytes in the medial striatum (*8*), and lower expression of several astrocytic genes such as *Agt*, *Sparc* and *Nnat*. Moreover, we reanalyzed scRNA-seq data generated simultaneously in cortical and striatal astrocytes (*39*). This confirmed that striatal astrocytes are enriched in *Cd9* and *Cd81* compared to cortical astrocytes, and thus that the differences that led to the classification of qNSC1 as RG-like cells might be linked with heterogeneity among astrocytes instead (Fig.S4D). Thus, we concluded that cells originally classified as deeply quiescent RG-like cells are in fact a population of astrocytes from the striatum, likely enriched medially and which emerges between P3 and P90.

A recent multi-omics study including qNSC1 and qNSC2 was recently published (*38*), in which the authors conclude that the methylome of the cells classified as qNSC1 was closer to that of control astrocytes than to that of RG-like cells. Although they renamed them "vSVZ astrocytes" they also proposed that these cells might be B1 cells, which are RG-like cells. A recent report characterized both B1 and B2 cells in the SEZ (*45*), and the markers identified as labelling them belong to the cells that were previously classified as qNSC2 and which can also be mapped throughout multiple datasets according to our analyses. Thus, so-called qNSC1 and qNSC2 can be reliably distinguished not only via their methylome but also via their transcriptome, and qNSC1 cells are actually astrocytes while qNSC2 cells are bona fide RG-like cells. Importantly this does not call into question the functional properties that had been identified for these cells, except for the interpretation that qNSC1 cells reactivate by becoming "qNSC2" cells. Indeed, we consider it highly unlikely that parenchymal cells turn into RG-like cells. Instead it is more likely that they go through a state with a transcriptome similar to that of NSCs while remaining parenchymal astrocytes, as had been previously postulated (*38*). Overall this interpretation reconciles multiple studies on the SEZ with hitherto diverging results, unifies the description of reactive neurogenesis and is supported by methylomic as well as both regular and spatial (*42*) transcriptomic data and consistent with in situ validation with RNAScope (*45*). For these reasons, we believe that the initial

distinction between qNSC2 and qNSC1 should instead be treated as a distinction between RG-like cells and local astrocytes, respectively, the latter likely being the same population as the population of medial striatal astrocytes previously identified as latent NSCs, which is how we considered them in our final analyses.

# Supplementary Figures and Legends

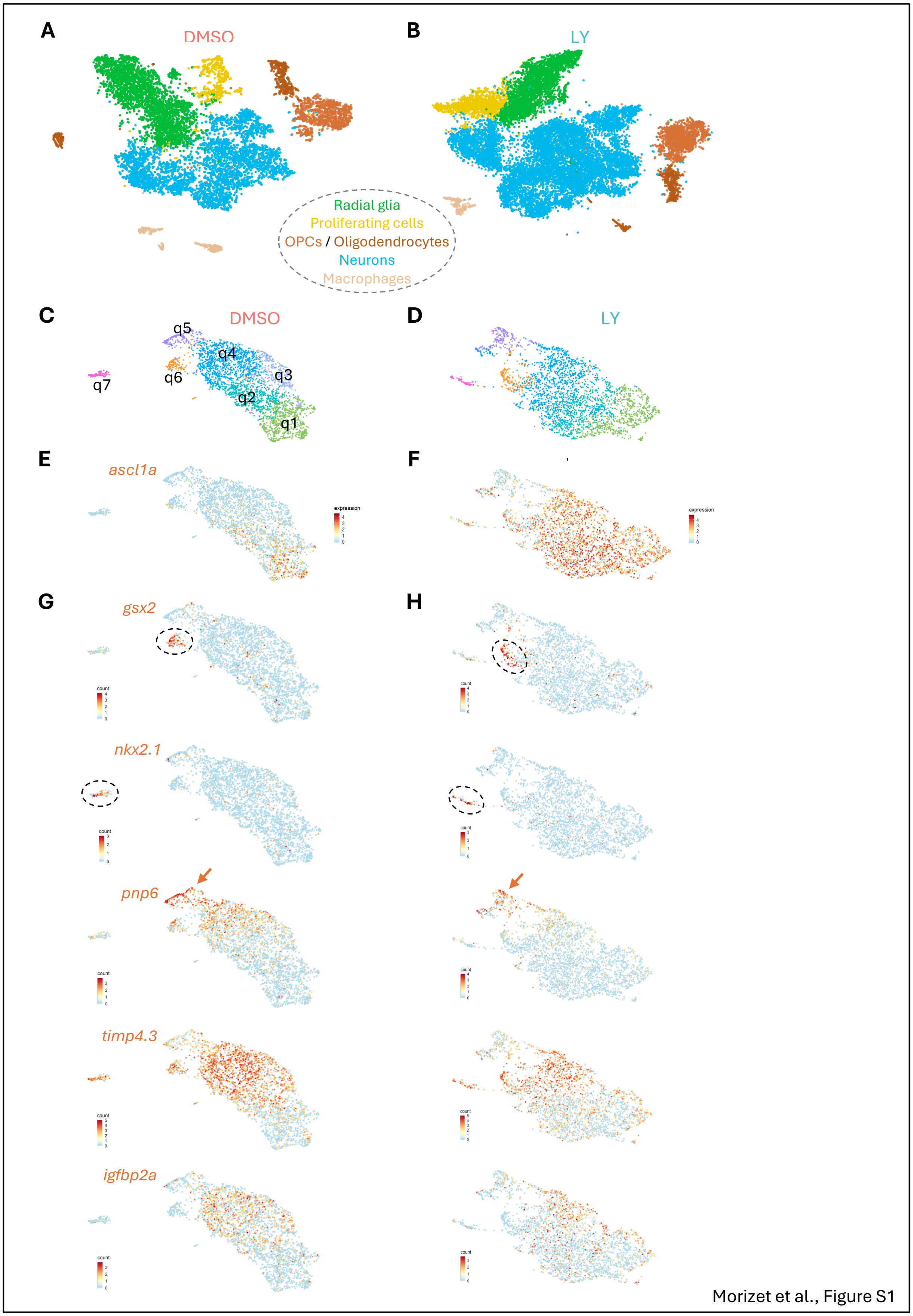

**Figure S1 - *ascl1a* is upregulated but major qNSC subpopulation markers display similar patterns following 24 hours of DMSO and LY treatment. A,B.** tSNE of all cells of the DMSO (A) and LY (B) treatment scRNAseq datasets colored by their broad cell type annotation (Table S1, as in (*16*)). Cells selected as qNSCs (green) and proliferating cells (yellow) are color coded. Abbreviations: a: activated; q: quiescent. **C.** Low dimensional embedding of qNSCs in a DMSO control scRNAseq dataset colored by cluster (same as in Fig.1H, from (*16*))**. D.** Low dimensional embedding of qNSCs in the LY-treated scRNAseq dataset colored by cluster (as in Fig.1I)**. E,F.** Levels of expression of *ascl1a* in the DMSO (C) and LY (D) datasets. **G.** Levels of expression of cluster-indicating genes in the control dataset. Rare clusters identified by *nkx2.1* (q7) and *gsx2* (q6) are circled to highlight them, and q5 is indicated by an arrow. **H.** Levels of expression of the same genes in the LY-treated dataset.

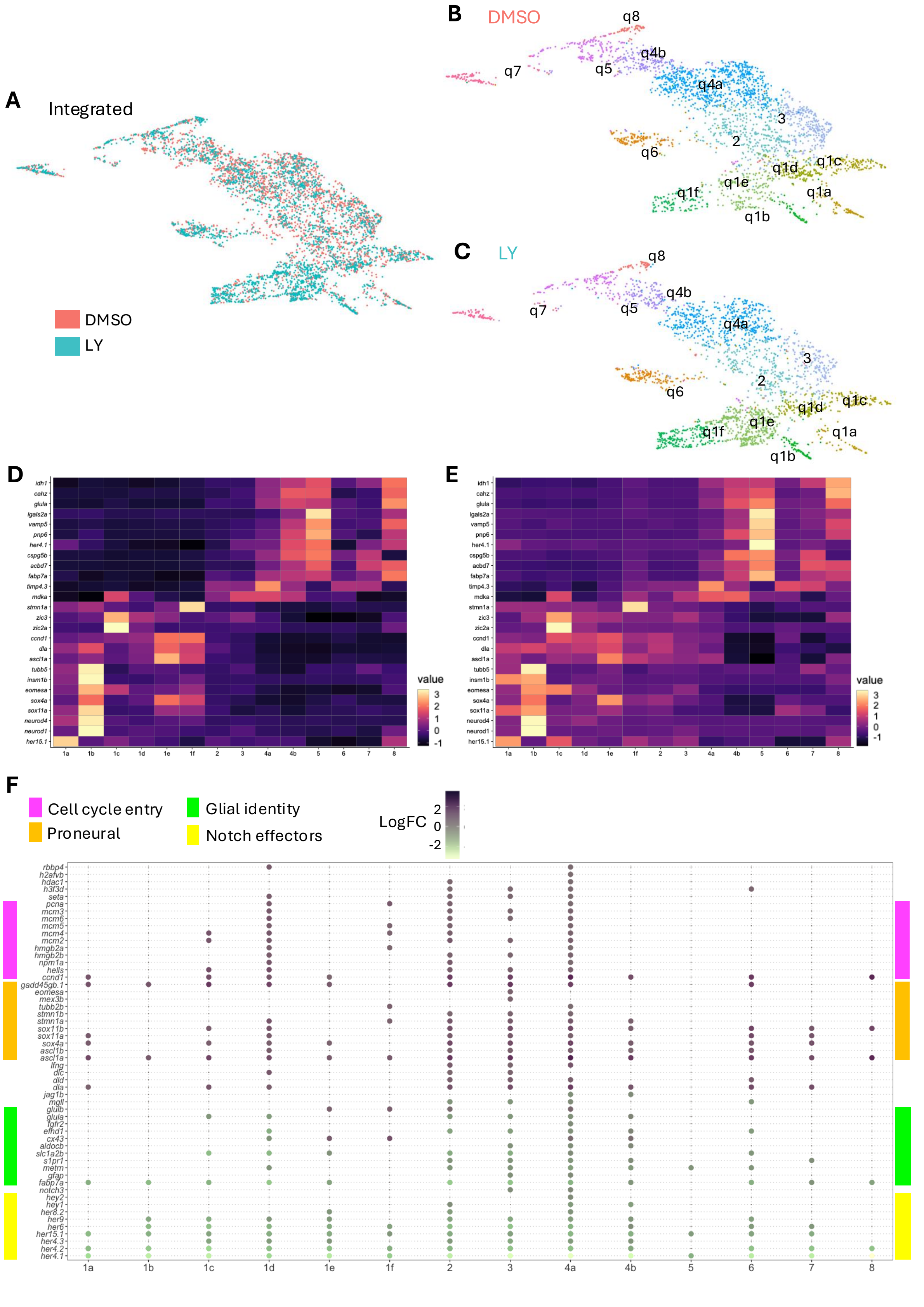


Morizet et al., Figure S2

**Figure S2 - Integrative analysis highlights novel qNSC subclusters and differentially expressed genes between LY-treated and untreated qNSCs in each cluster**. **A.** Low dimensional embedding of the joint qNSCs scRNAseq dataset colored by condition (control in red and LY-treated in blue). **B,C.** Projections of individual datasets (B: DMSO, C: LY) on the low-dimensional embedding color-coded by cluster. This highlights that the two datasets thoroughly mix but that several clusters remain clearly identifiable. **D,E.** Heatmaps showing, for a selection of genes, relative mean gene expression per cluster in the DMSO (D) and LY (E) datasets. Because expression levels differ between genes, a scaling per gene was applied (for each gene, an expression level equal to the mean of expression in all clusters is set to zero, and clusters with expression higher or lower than the mean are positive or negative, respectively). **F.** Dotplot of a subset of differentially expressed genes between LY-treated and untreated cells in each qNSC cluster. A subset of genes known to be Notch targets (among which Notch effectors are highlighted in yellow) and/or associated with glial identity (highlighted in green), proneural activity (highlighted in orange), cell cycle entry (highlighted in pink) or chromatin remodeling (other genes) was chosen for visualization. Dot size is binary based on whether the adjusted pvalue of the MAST differential gene expression test with nUMI as a latent variable is below 0.05 or not. Dot color is determined by log2 fold change, with purple indicating upregulation in LY-treated cells and green indicating downregulation in LY-treated cells.

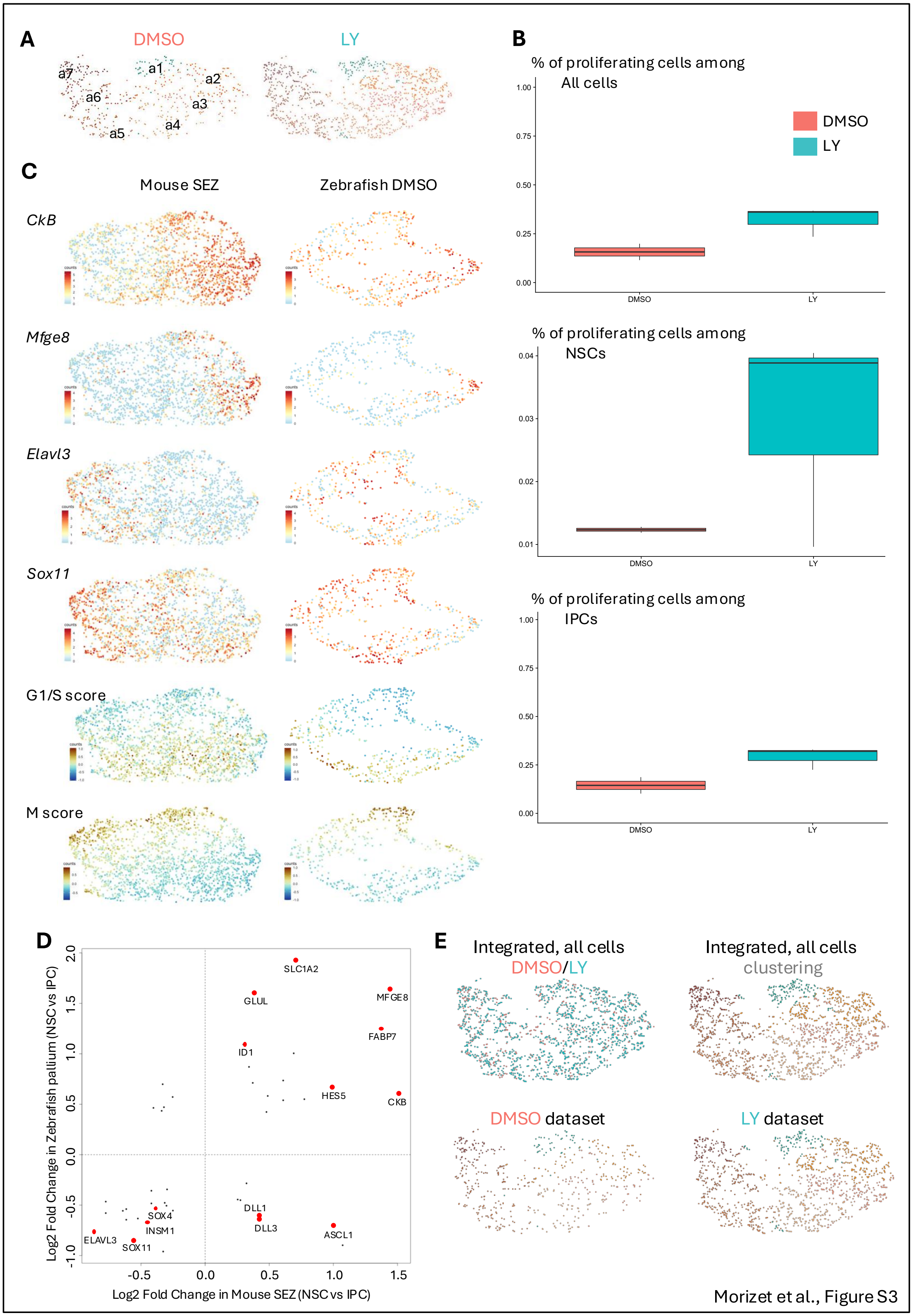


**Figure S3 - Analysis of cycling cells in the adult mouse SEZ and zebrafish pallium reveals comparable NSCs, IPCs and cell cycle phases. A.** Projection of the cycling cells of each individual

zebrafish pallial dataset (DMSO-treated, top (*16*), and LY-treated, bottom -this study-) on the integrated embedding of cycling cells, color-coded by cluster derived from integrated analysis. Note the higher total number of cells in the LY-treated dataset. **B.** Percentage of proliferating cells per cell category (from top to bottom: all cells, NSCs and IPCs) in the DMSO versus LY datasets (color-coded). **C.** Low dimensional embeddings of cycling cells from the mouse SEZ (left) (from (*13*)) and zebrafish pallium (right) (from (*16*)) colored by levels of expression of genes enriched in cycling NSCs (*Ckb*, *Mfge8*) or IPCs (*Elavl3*, *Sox11*) and cell cycle scores. Color coding is distinct to separate genes from scores. In both cases two independent sources of variation can be identified transcriptionally and visualized on the lower dimensional embedding: an axis (horizontal) separating NSCs from IPCs and an axis (vertical) going from cells in early phases of the cell cycle to cells in late phases of the cell cycle. **D.** Plot of log2 Fold Change in zebrafish NSCs over IPCs in relation to log2 Fold Change in murine NSCs over IPCs after conversion to a common set of orthologs. A majority of genes that are detected as significant in both datasets fall in correlated quadrants. Among them, *Hes5* is the only effector of the Notch pathway with significantly higher expression in NSCs than in IPCs in both zebrafish and mice. **E.** Low dimensional embedding of the joint scRNAseq datasets of cycling cells for the DMSO- and LY-treated conditions, color-coded by condition (top left) (DMSO: red; LY-treated: cyan), by cluster (top right) (the two datasets thoroughly mix but)several clusters remain clearly identifiable., or showing only the cells of the DMSO dataset (bottom left) or the cells of the LY dataset (bottom right).

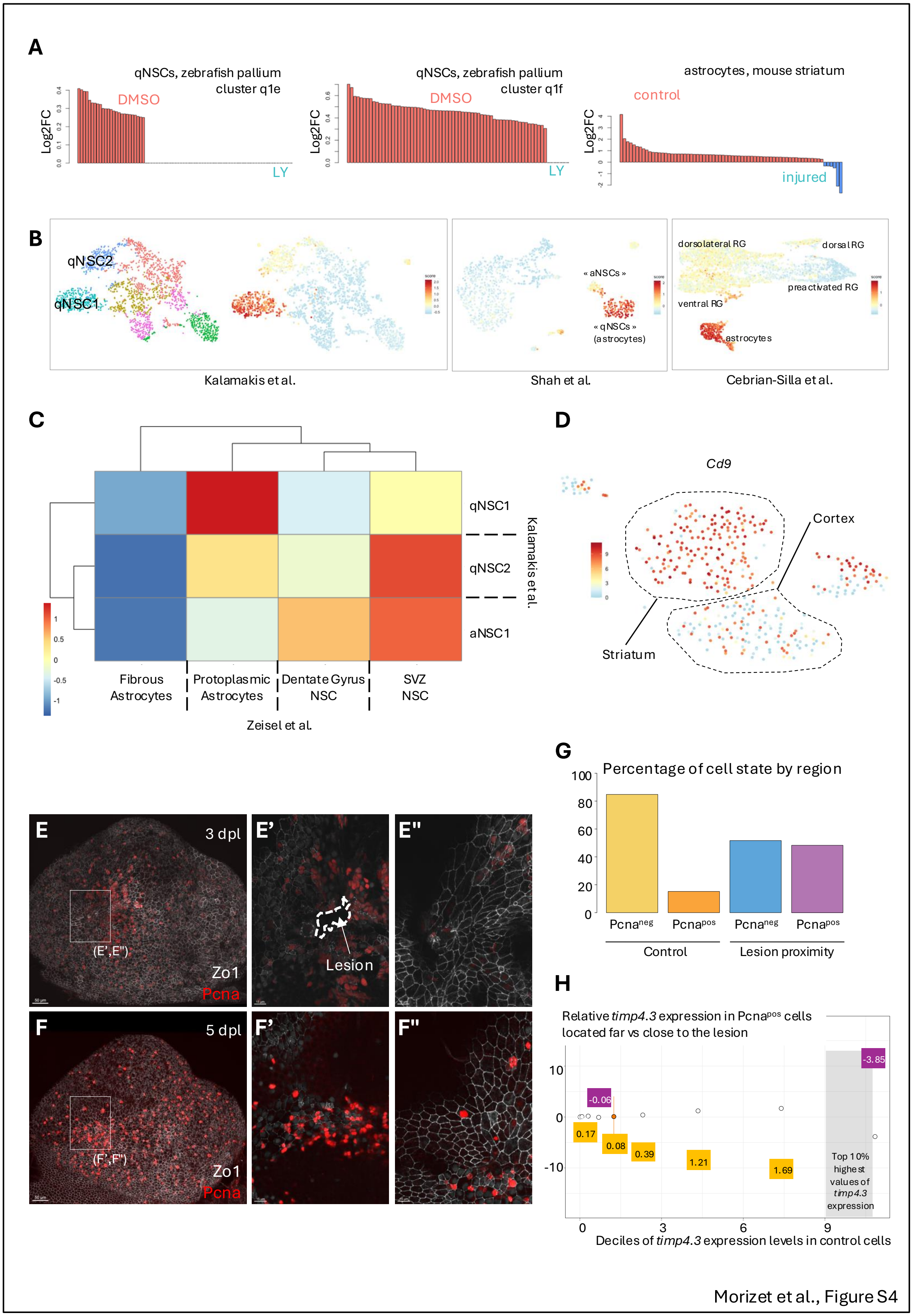


**Figure S4 - Zebrafish astrocyte-like NSCs and murine latent NSCs share features when forcefully reactivated by Notch inhibition or injury. A.** Left and middle panels: log2 Fold Change

(log2FC) of the expression of ribosomal genes detected as significantly differentially expressed between LY-treated and untreated cells in clusters q1e (left) and q1f (middle). Cell transition to q1e and q1f upon LY treatment is not accompanied with a normal upregulation of ribosomal genes. Right panel: log2 Fold Change of the expression of ribosomal genes between cells found in "qNSC2" (see Fig.S4B) after injury and unperturbed "qNSC2" cells (*12*). Ribosomal genes were not sorted for statistical significance in this case because of the low power of statistical tests with as few cells as were profiled in these scRNAseq experiments with Smart-seq technology. **B-D.** Reanalysis of mouse scRNAseq datasets profiling latent neural stem cells, variably classified as dormant NSCs (qNSC1 in (*12*)), as the only qNSCs (*41*) or as astrocytes (*8*). Detailed reanalysis of these datasets, mapping to a murine telencephalon atlas encompassing both NSCs and astrocytes (*44*) and the use of specific markers that we previously identified as being reliable to distinguish between astrocytes and NSCs across neurogenic niches (*16*) suggested that they all correspond to the same subpopulation of mature medial striatal astrocytes. **B.** Identification and projection of a score specifically identifying qNSC1 (*12*) cells to other adult mouse SEZ datasets (*8*, *41*). The cells displaying highest qNSC1 scores are putative astrocytes from these other datasets (see also Methods, and new conclusions on qNSC1 in (*38*)). **C.** Metaneighbour (*43*) mapping of non-proliferating astroglia between a SEZ dataset describing the presence of qNSC1 cells (*11*) and astroglia from the telencephalon extracted from a whole brain cell atlas (*44*). Contrary to qNSC2 and aNSC1, qNSC1 are closer to protoplasmic astrocytes than to RG-like cells. **D.** Compared expression of *Cd9*, a putative marker of qNSC1 between striatal and cortical astrocytes in the adult mouse (*39*). **E,F.** Example images of lesioned pallia at 3dpl (E) or 5dpl (F). E, F are Images represent large fields of view of 3D whole-mount hemispheres illustrating localized increase in proliferation around the lesion. Scale bar: 50µm. E′-F″ are close up views of ≈10µm thickness centered on the lesions at different depths. Scale bars: 15µm. E′, F′ are at the parenchymal depth where the lesion is best visible; E″, F″ are at the ventricular surface, showing the tela choroida, which was not removed. An abundant proliferative response can be observed at 3dpl and 5dpl, specifically in the NSC layer and just below (E′, F′). At 3dpl the lesion itself is still visible whereas at 5dpl the hole has been filled. **G.** Percentage of Pcna$^{neg}$ and Pcna$^{pos}$ cells in ventricular regions located far from the lesion (control area) or close to the lesion (lesion proximity). Cells close to the lesion proliferate more, as expected with a regenerative response. **H.** Relative *timp4.3* expression in Pcna$^{pos}$ cells between cells close to the lesion (control cells) vs far from the lesion (y axis) as a function of the

decile of *timp4.3* expression in control cells (x axis). Representation of the shift function as calculated in (*82*). The x-axis represents *timp4.3* expression values, measured as number of dots in smFISH, in control cells binned by deciles and the y-axis represents the difference between the average *timp4.3* expression value in cells neighboring the lesion and in control cells for each decile of their respective distribution. The circles represent the point estimates of this difference for each decile and the vertical bars represent the confidence interval. A positive difference suggests that the average *timp4.3* expression is higher in the control cells than in cells neighboring the lesion. The top 10% values of *timp4.3* expression (gray area) appear on average higher in cells close to the lesion than in cells far away from the lesion, but the difference is not statistically significant.

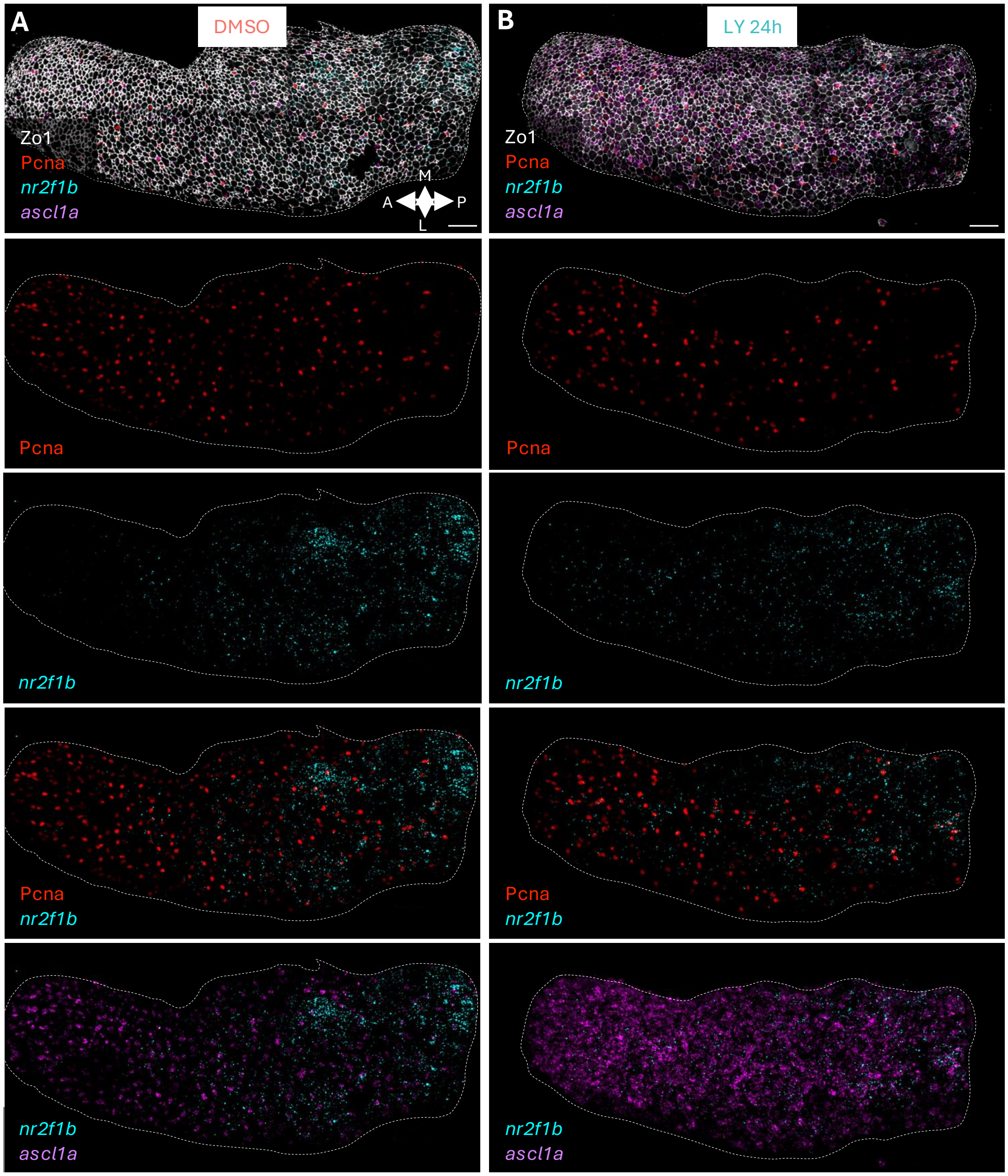


Morizet et al., Figure S5

**Figure S5 - *nr2f1b* and *ascl1a* are expressed in opposite gradients in the dorsal pallium and this prefigures the resistance to LY treatment.** Whole-mount dorsal view of a telencephalic

hemisphere (anterior left) in fish treated with DMSO or LY for 24 hours and immunostained for ZO1 (white, apical junctions) and Pcna (red, proliferation) and processed for smFISH for *nr2f1b* (cyan) and *ascl1a* (magenta). *ascl1a* and Pcna expression are enriched rostrally in the dorsal pallium in control conditions whereas *nr2f1b* expression is enriched caudally. After 24 hours of LY treatment, *ascl1a* expression is more widespread but remains enriched rostrally (this also applies to Pcna after 48 hours, see Fig.S6A middle panel), opposite to *nr2f1b*. Scale bars: 50μm.

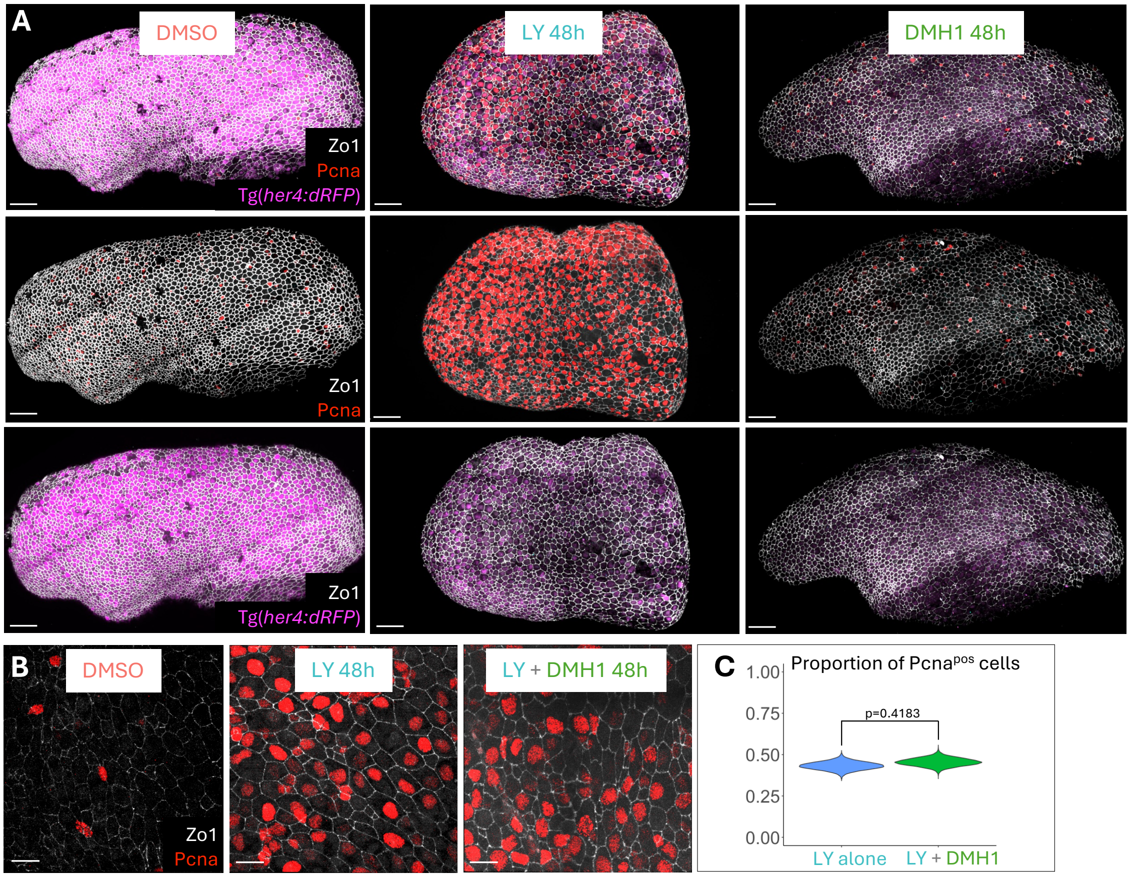


Morizet et al., Figure S6

**Figure S6: Assessment of putative regulatory mechanisms explaining the resistance to Notch inhibition. A.** Confocal images of whole individual telencephalic hemispheres from

Tg(*her4*:dRFP) fish treated for 48 hours with either DMSO (left panels), LY (middle) or DMH1 (right). The brains were immunostained for ZO1 (white, apical junctions), Pcna (red, proliferation) and dRFP (magenta). The top row shows all channels, the middle row shows the detection of ZO1 and RFP, and the bottom row shows ZO1 and Pcna. While both LY and DMH1 efficiently decrease dRFP, only LY increases proliferation. Scale bars: 50 µm. **B.** Confocal images of caudal areas in the dorsal pallium of fish treated for 48 hours with either DMSO (left panel), LY alone (middle) or LY and DMH1 together (right), and immunostained for ZO1 (white, apical junctions) and Pcna (red, proliferation). Scale bars: 15µm. **C.** Quantification of the association between type of treatment and likelihood to start cycling after 48 hours of treatment. No significant difference is detected between treatments with LY alone or LY + DMH1. Reported p-value is derived from a chi square test. Violin plots are built from bootstrapped random sampling of the measured proportions to estimate the distribution.

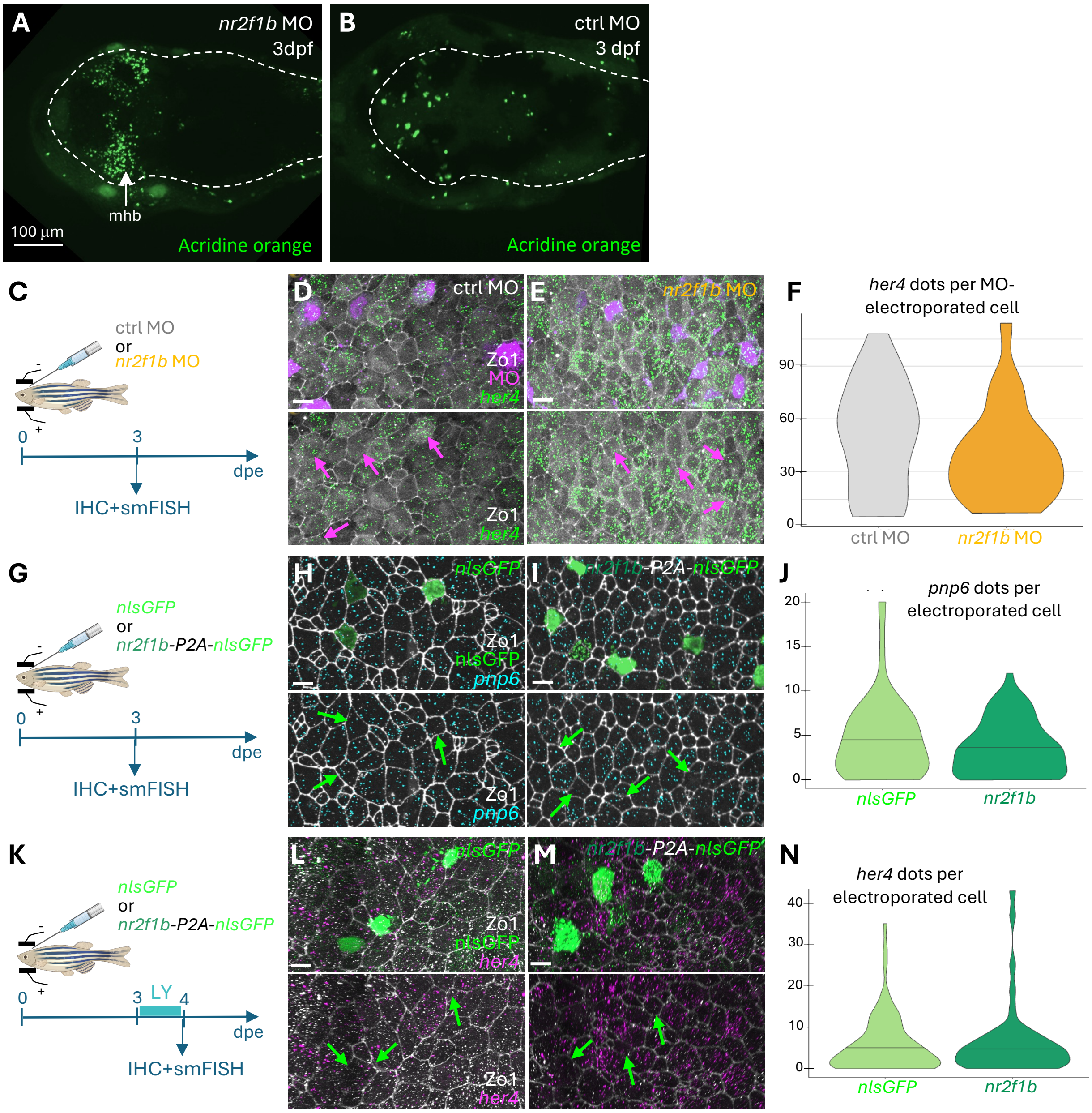


Morizet et al., Figure S7

209

**Figure S7. Further validations of genetic tools and output of functional *nr2f1b* manipulations. A,B.** Validation of the efficiency and specificity of the *nr2f1b* MO. Cell death was monitored using Acridine orange (green) at 3dpf following MO injection at the 1-cell stage. Dorsal views of whole embryos, anterior left; the brains are surrounded by dotted lines, and the midbrain-hindbrain boundary (mhb) is indicated. *nr2f1b* MO-injected embryos at 3dpf display cell death at the mhb, mimicking *nr2f1b* homozygous null mutants (*60*). **C-F.** *nr2f1b* knock-down and expression of *her4*. **C.** Schematic of the experiment. After morpholino (MO) intracranial injection and electroporation, fish were allowed to rest for 3 days, then sacrificed for quantification of *her4* expression. **D,E.** Example images of the pallial surface in fish electroporated with a control MO (D) and the *nr2f1b*-specific MO (E) as in C (dorsal whole-mount views). The brains were processed for smFISH for *her4* (green) and immunohistochemistry for Zo1 (white), and the lissamine-tagged MOs (magenta) were imaged directly. Bottom panels omit the MO channel but arrows point to electroporated cells. Scale bars: 10µm. **F.** Corresponding quantifications. Statistics : unpaired two-sample Wilcoxon-test, p-value=0.2006; 3 hemispheres per condition, n=48 cells. **G-J.** *nr2f1b* overexpression and expression of *pnp6*. **G.** Schematic of the experiment. After plasmid DNA intracranial injection and electroporation, fish were allowed to rest for 3 days, then sacrificed for quantification of *pnp6* expression. **H,I.** Example images of the pallial surface in fish electroporated with a control nlsGFP plasmid (H) and the *nr2f1b*-P2A-nlsGFP overexpression plasmid (I) as in G (dorsal whole-mount views). The brains were processed for smFISH for *pnp6* (cyan) and immunohistochemistry for Zo1 (white) and GFP (green). Bottom panels omit the GFP channel but arrows point to electroporated cells. Scale bars: 10µm. **J.** Corresponding quantifications. Statistics: unpaired two-sample Wilcoxon-test, p-value=0.28; 3 hemispheres per condition, n=123 cells. **K-N.** *nr2f1b* overexpression and expression of *her4*. **K.** Schematic of the experiment. After plasmid DNA intracranial injection and electroporation, fish were allowed to rest for 3 days, then treated with LY for 1 day and sacrificed for quantification of *her4* expression. **L,M.** Example images of the pallial surface in fish electroporated with a control nlsGFP plasmid (L) and the *nr2f1b*-P2A-nlsGFP overexpression plasmid (M) as in K (dorsal whole-mount views). The brains were processed for smFISH for *her4* (green) and immunohistochemistry for Zo1 (white) and GFP (green). Bottom panels omit the GFP channel but arrows point to electroporated cells. Scale bars: 10µm. **N.** Corresponding quantifications. Statistics: unpaired two-sample Wilcoxon-test, p-value=0.84; 3 hemispheres per condition, n=115 cells.

# Supplementary Tables and Legends

| Broad cluster |
|---|
| Radial Glia |
| Neurons (different types and at different levels of maturation) |
| Proliferating cells (mostly aNSCs, IPCs, and a few proliferating oligodendrocytes that could be remove |
| Oligodendrocytic lineage |
| Macrophage lineage |

**Table S1. Marker genes used to define broad cell identities from the full scRNAseq dataset.**

Table S2

| Cluster | % of ccnd1+ cells |
|---|---|
| DMSO_1a | 59.4 |
| LY_1a | 96 |
| DMSO_1b | 71.4 |
| LY_1b | 87.9 |
| DMSO_1c | 65.3 |
| LY_1c | 97.4 |
| DMSO_1d | 74.7 |
| LY_1d | 97.1 |
| DMSO_1e | 87 |
| LY_1e | 96.8 |
| DMSO_1f | 88.8 |
| LY_1f | 94.7 |
| DMSO_2 | 45.7 |
| LY_2 | 90.7 |
| DMSO_3 | 35.3 |
| LY_3 | 87.9 |
| DMSO_4a | 18.9 |
| LY_4a | 86.8 |
| DMSO_4b | 13.3 |
| LY_4b | 55.3 |
| DMSO_5 | 10.8 |
| LY_5 | 15.3 |
| DMSO_6 | 23.5 |
| LY_6 | 73.4 |
| DMSO_7 | 12.6 |
| LY_7 | 33.3 |
| DMSO_8 | 2.9 |
| LY_8 | 41.9 |

**Table S2. Proportion of cells expressing *ccnd1* in each cluster under DMSO and LY conditions.**

Table S3

| Gene name | Entrez Gene ID | Accession Number | Channel |
|---|---|---|---|
| *nr2f1b* | 393564 | NM_200592.1 | T2 |
| *ascl1a* | 30466 | NM_131219.1 | T1 |
| *ascl1a* | 30466 | NM_131219.1 | T3 |
| *timp4.3* | 100124610 | ENSDART00000187110.1 | T2 |
| *her4* | 100149863 | NM_001103128.1 | T1 |
| *her4* | 100149863 | NM_001103128.1 | T5 |
| *ccnd1* | 30222 | NM_131025.4 | T3 |

**Table S3. RNAscope probes used in this study**

## Legends for Data S1-S3

**Data S1. Differentially expressed genes (DEGs) between DMSO and LY conditions in each cluster of quiescent NSCs/IPCs.** Positive LogFC when expression is higher in the DMSO dataset. pctDMSO, pctLY: proportion of cells expressing the gene in the DMSO and LY datasets, respectively.

**Data S2. Differentially expressed genes (DEGs) between DMSO and LY conditions in each cluster of proliferating NSCs/IPCs.** Positive LogFC when expression is higher in the DMSO dataset. pctDMSO, pctLY: proportion of cells expressing the gene in the DMSO and LY datasets, respectively.

**Data S3. Antibodies used in this study**

## REFERENCES AND NOTES


1. N. Urbán, D. L. C. van den Berg, A. Forget, J. Andersen, J. A. A. Demmers, C. Hunt, O. Ayrault, F. Guillemot, Return to quiescence of mouse neural stem cells by degradation of a proactivation protein. *Science* **353**, 292–295 (2016).

2. S. Bottes, B. N. Jaeger, G.-A. Pilz, D. J. Jörg, J. D. Cole, M. Kruse, L. Harris, V. I. Korobeynyk, I. Mallona, F. Helmchen, F. Guillemot, B. D. Simons, S. Jessberger, Long-term self-renewing stem cells in the adult mouse hippocampus identified by intravital imaging. *Nat. Neurosci.* **24**, 225–233 (2021).

3. A. Ibrayeva, M. Bay, E. Pu, D. J. Jörg, L. Peng, H. Jun, N. Zhang, D. Aaron, C. Lin, G. Resler, A. Hidalgo, M.-H. Jang, B. D. Simons, M. A. Bonaguidi, Early stem cell aging in the mature brain. *Cell Stem Cell* **28**, 955–966.e7 (2021).

4. J. P. Magnusson, C. Göritz, J. Tatarishvili, D. O. Dias, E. M. K. Smith, O. Lindvall, Z. Kokaia, J. Frisén, A latent neurogenic program in astrocytes regulated by Notch signaling in the mouse. *Science* **346**, 237–241 (2014).

5. E. Than-Trong, B. Kiani, N. Dray, S. Ortica, B. Simons, S. Rulands, A. Alunni, L. Bally-Cuif, Lineage hierarchies and stochasticity ensure the long-term maintenance of adult neural stem cells. *Sci. Adv.* **6**, eaaz5424 (2020).

6. L. Mancini, B. Guirao, S. Ortica, M. Labusch, F. Cheysson, V. Bonnet, M. S. Phan, S. Herbert, P. Mahou, E. Menant, S. Bedu, J.-Y. Tinevez, C. Baroud, E. Beaurepaire, Y. Bellaiche, L. Bally-Cuif, N. Dray, Apical size and *deltaA* expression predict adult neural stem cell decisions along lineage progression. *Science* **9**, eadg7519 (2023).

7. A. Alunni, M. Krecsmarik, A. Bosco, S. Galant, L. Pan, C. B. Moens, L. Bally-Cuif, Notch3 signaling gates cell cycle entry and limits neural stem cell amplification in the adult pallium. *Development* **140**, 3335–3347 (2013).

8. A. Cebrian-Silla, M. A. Nascimento, S. A. Redmond, B. Mansky, D. Wu, K. Obernier, R. Romero Rodriguez, S. Gonzalez-Granero, J. M. García-Verdugo, D. A. Lim, A. Álvarez-

Buylla, Single-cell analysis of the ventricular-subventricular zone reveals signatures of dorsal and ventral adult neurogenesis. *eLife* **10**, e67436 (2021).

9. L. Harris, P. Rigo, T. Stiehl, Z. B. Gaber, S. H. L. Austin, M. del Mar Masdeu, A. Edwards, N. Urbán, A. Marciniak-Czochra, F. Guillemot, Coordinated changes in cellular behavior ensure the lifelong maintenance of the hippocampal stem cell population. *Cell Stem Cell* **28**, 863–876.e6 (2021).

10. H. Hochgerner, A. Zeisel, P. Lönnerberg, S. Linnarsson, Conserved properties of dentate gyrus neurogenesis across postnatal development revealed by single-cell RNA sequencing. *Nat. Neurosci.* **21**, 290–299 (2018).

11. G. Kalamakis, D. Brüne, S. Ravichandran, J. Bolz, W. Fan, F. Ziebell, T. Stiehl, F. Catalá-Martinez, J. Kupke, S. Zhao, E. Llorens-Bobadilla, K. Bauer, S. Limpert, B. Berger, U. Christen, P. Schmezer, J. P. Mallm, B. Berninger, S. Anders, A. del Sol, A. Marciniak-Czochra, A. Martin-Villalba, Quiescence modulates stem cell maintenance and regenerative capacity in the aging brain. *Cell* **176**, 1407–1419.e14 (2019).

12. E. Llorens-Bobadilla, S. Zhao, A. Baser, G. Saiz-Castro, K. Zwadlo, A. Martin-Villalba, Single-cell transcriptomics reveals a population of dormant neural stem cells that become activated upon brain injury. *Cell Stem Cell* **17**, 329–340 (2015).

13. D. Mizrak, H. M. Levitin, A. C. Delgado, V. Crotet, J. Yuan, Z. Chaker, V. Silva-Vargas, P. A. Sims, F. Doetsch, Single-cell analysis of regional differences in adult V-SVZ neural stem cell lineages. *Cell Rep.* **26**, 394–406.e5 (2019).

14. J. Shin, D. A. Berg, Y. Zhu, J. Y. Shin, J. Song, M. A. Bonaguidi, G. Enikolopov, D. W. Nauen, K. M. Christian, G.-l. Ming, H. Song, Single-cell RNA-seq with waterfall reveals molecular cascades underlying adult neurogenesis. *Cell Stem Cell* **17**, 360–372 (2015).

15. V. Zywitza, A. Misios, L. Bunatyan, T. E. Willnow, N. Rajewsky, Single-cell transcriptomics characterizes cell types in the subventricular zone and uncovers molecular defects impairing adult neurogenesis. *Cell Rep.* **25**, 2457–2469.e8 (2018).

16. D. Morizet, I. Foucher, A. Alunni, L. Bally-Cuif, Reconstruction of macroglia and adult neurogenesis evolution through cross-species single-cell transcriptomic analyses. *Nat. Commun.* **15**, 3306 (2024).

17. L. Anneser, C. Satou, H.-R. Hotz, R. W. Friedrich, Molecular organization of neuronal cell types and neuromodulatory systems in the zebrafish telencephalon. *Curr. Biol.* **34**, 298–312.e4 (2024).

18. P. P. D'Gama, T. Qiu, M. I. Cosacak, D. Rayamajhi, A. Konac, J. N. Hansen, C. Ringers, F. Acuña-Hinrichsen, S. P. Hui, E. W. Olstad, Y. L. Chong, C. K. A. Lim, A. Gupta, C. P. Ng, B. S. Nilges, N. D. Kashikar, D. Wachten, D. Liebl, K. Kikuchi, C. Kizil, E. Yaksi, S. Roy, N. Jurisch-Yaksi, Diversity and function of motile ciliated cell types within ependymal lineages of the zebrafish brain. *Cell Rep.* **37**, 109775 (2021).

19. M. I. Cosacak, P. Bhattarai, S. Reinhardt, A. Petzold, A. Dahl, Y. Zhang, C. Kizil, Single-cell transcriptomics analyses of neural stem cell heterogeneity and contextual plasticity in a zebrafish brain model of amyloid toxicity. *Cell Rep.* **27**, 1307–1318.e3 (2019).

20. N. Mitic, A. Neuschulz, B. Spanjaard, J. Schneider, N. Fresmann, K. T. Novoselc, T. Strunk, L. Münster, P. Olivares-Chauvet, J. Ninkovic, J. P. Junker, Dissecting the spatiotemporal diversity of adult neural stem cells. *Mol. Syst. Biol.* **20**, 321–337 (2024).

21. Y. Harada, M. Yamada, I. Imayoshi, R. Kageyama, Y. Suzuki, T. Kuniya, S. Furutachi, D. Kawaguchi, Y. Gotoh, Cell cycle arrest determines adult neural stem cell ontogeny by an embryonic Notch-nonoscillatory Hey1 module. *Nat. Commun.* **12**, 6562 (2021).

22. O. Basak, C. Giachino, E. Fiorini, H. R. MacDonald, V. Taylor, Neurogenic subventricular zone stem/progenitor cells are Notch1-dependent in their active but not quiescent state. *J. Neurosci.* **32**, 5654–5666 (2012).

23. E. Than-Trong, S. Ortica-Gatti, S. Mella, C. Nepal, A. Alunni, L. Bally-Cuif, Neural stem cell quiescence and stemness are molecularly distinct outputs of the Notch3 signalling cascade in the vertebrate adult brain. *Development* **145**, dev161034 (2018).

24. P. Chapouton, P. Skupien, B. Hesl, M. Coolen, J. C. Moore, R. Madelaine, E. Kremmer, T. Faus-Kessler, P. Blader, N. D. Lawson, L. Bally-Cuif, Notch activity levels control the balance between quiescence and recruitment of adult neural stem cells. *J. Neurosci.* **30**, 7961–7974 (2010).

25. A. Engler, C. Rolando, C. Giachino, I. Saotome, A. Erni, C. Brien, R. Zhang, U. Zimber-Strobl, F. Radtke, S. Artavanis-Tsakonas, A. Louvi, V. Taylor, Notch2 signaling maintains NSC quiescence in the murine ventricular-subventricular zone. *Cell Rep.* **22**, 992–1002 (2018).

26. R. Zhang, M. Boareto, A. Engler, A. Louvi, C. Giachino, D. Iber, V. Taylor, Id4 downstream of Notch2 maintains neural stem cell quiescence in the adult hippocampus. *Cell Rep.* **28**, 1485–1498.e6 (2019).

27. R. Sueda, I. Imayoshi, Y. Harima, R. Kageyama, High Hes1 expression and resultant Ascl1 suppression regulate quiescent vs. active neural stem cells in the adult mouse brain. *Genes Dev.* **33**, 511–523 (2019).

28. J. Andersen, N. Urbán, A. Achimastou, A. Ito, M. Simic, K. Ullom, B. Martynoga, M. Lebel, C. Göritz, J. Frisén, M. Nakafuku, F. Guillemot, A transcriptional mechanism integrating inputs from extracellular signals to activate hippocampal stem cells. *Neuron* **83**, 1085–1097 (2014).

29. J. Shin, J. Chen, L. Solnica-Krezel, Efficient homologous recombination-mediated genome engineering in zebrafish using TALE nucleases. *Development* **141**, 3807–3818 (2014).

30. J. Liu, C. Gao, J. Sodicoff, V. Kozareva, E. Z. Macosko, J. D. Welch, Jointly defining cell types from multiple single-cell datasets using LIGER. *Nat. Protoc.* **15**, 3632–3662 (2020).

31. M.-C. Tiveron, C. Beclin, S. Murgan, S. Wild, A. Angelova, J. Marc, N. Coré, A. de Chevigny, E. Herrera, A. Bosio, V. Bertrand, H. Cremer, Zic-proteins are repressors of dopaminergic forebrain fate in mice and *C. elegans*. *J. Neurosci.* **37**, 10611–10623 (2017).

32. S. I. A. Bukhari, S. Vasudevan, FXR1a-associated microRNP: A driver of specialized non-canonical translation in quiescent conditions. *RNA Biol.* **14**, 137–145 (2017).

33. J. Nishino, K. Yamashita, H. Hashiguchi, H. Fujii, T. Shimazaki, H. Hamada, Meteorin: A secreted protein that regulates glial cell differentiation and promotes axonal extension. *EMBO J.* **23**, 1998–2008 (2004).

34. J. J. Breunig, J. Silbereis, F. M. Vaccarino, N. Šestan, P. Rakic, Notch regulates cell fate and dendrite morphology of newborn neurons in the postnatal dentate gyrus. *Proc. Natl. Acad. Sci. U.S.A.* **104**, 20558–20563 (2007).

35. A. Santos, R. Wernersson, L. J. Jensen, Cyclebase 3.0: A multi-organism database on cell-cycle regulation and phenotypes. *Nucleic Acids Res.* **43**, D1140–D1144 (2015).

36. N. Urbán, I. M. Blomfield, F. Guillemot, Quiescence of adult mammalian neural stem cells: A highly regulated rest. *Neuron* **104**, 834–848 (2019).

37. P. Kaldis, Quo Vadis cell growth and division? *Front. Cell Dev. Biol.* **4**, (2016).

38. L. P. M. Kremer, S. Cerrizuela, H. El-Sammak, M. E. Al Shukairi, T. Ellinger, J. Straub, A. Korkmaz, K. Volk, J. Brunken, S. Kleber, S. Anders, A. Martin-Villalba, DNA methylation controls stemness of astrocytes in health and ischaemia. *Nature* **634**, 415–423 (2024).

39. J. P. Magnusson, M. Zamboni, G. Santopolo, J. E. Mold, M. Barrientos-Somarribas, C. Talavera-Lopez, B. Andersson, J. Frisén, Activation of a neural stem cell transcriptional program in parenchymal astrocytes. *eLife* **9**, e59733 (2020).

40. M. J. Borrett, B. T. Innes, D. Jeong, N. Tahmasian, M. A. Storer, G. D. Bader, D. R. Kaplan, F. D. Miller, Single-cell profiling shows murine forebrain neural stem cells reacquire a developmental state when activated for adult neurogenesis. *Cell Rep.* **32**, 108022 (2020).

41. P. T. Shah, J. A. Stratton, M. G. Stykel, S. Abbasi, S. Sharma, K. A. Mayr, K. Koblinger, P. J. Whelan, J. Biernaskie, Single-cell transcriptomics and fate mapping of ependymal cells reveals an absence of neural stem cell function. *Cell* **173**, 1045–1057.e9 (2018).

42. G. Marcy, L. Foucault, E. Babina, E. Texeraud, S. Zweifel, C. Heinrich, H. Hernandez-Vargas, C. Parras, D. Jabaudon, O. Raineteau, Single cell analysis of the dorsal V-SVZ reveals differential quiescence of postnatal pallial and subpallial neural stem cells driven by

TGFβ/BMP-signalling. bioRxiv 492790 [Preprint] (2022). https://doi.org/10.1101/2022.05.20.492790.

43. M. Crow, A. Paul, S. Ballouz, Z. J. Huang, J. Gillis, Characterizing the replicability of cell types defined by single cell RNA-sequencing data using MetaNeighbor. *Nat. Commun.* **9**, 884 (2018).

44. A. Zeisel, H. Hochgerner, P. Lönnerberg, A. Johnsson, F. Memic, J. van der Zwan, M. Häring, E. Braun, L. E. Borm, G. La Manno, S. Codeluppi, A. Furlan, K. Lee, N. Skene, K. D. Harris, J. Hjerling-Leffler, E. Arenas, P. Ernfors, U. Marklund, S. Linnarsson, Molecular architecture of the mouse nervous system. *Cell* **174**, 999–1014.e22 (2018).

45. A. Cebrian-Silla, M. A. Nascimento, W. Mancia, S. Gonzalez-Granero, R. Romero-Rodriguez, K. Obernier, D. M. Steffen, D. A. Lim, J. M. Garcia-Verdugo, A. Alvarez-Buylla, Neural stem cell relay from b1 to b2 cells in the adult mouse ventricular-subventricular zone. bioRxiv 600695 [Preprint] (2024). https://doi.org/10.1101/2024.06.28.600695.

46. M. Lattke, R. Goldstone, J. K. Ellis, S. Boeing, J. Jurado-Arjona, N. Marichal, J. I. MacRae, B. Berninger, F. Guillemot, Extensive transcriptional and chromatin changes underlie astrocyte maturation in vivo and in culture. *Nat. Commun.* **12**, 4335 (2021).

47. M. März, R. Schmidt, S. Rastegar, U. Strähle, Regenerative response following stab injury in the adult zebrafish telencephalon. *Dev. Dyn.* **240**, 2221–2231 (2011).

48. T. Moerman, S. A. Santos, C. B. González-Blas, J. Simm, Y. Moreau, J. Aerts, S. Aerts, GRNBoost2 and Arboreto: Efficient and scalable inference of gene regulatory networks. *Bioinformatics* **35**, 2159–2161 (2019).

49. K. J. Webb, M. Coolen, C. J. Gloeckner, C. Stigloher, B. Bahn, S. Topp, M. Ueffing, L. Bally-Cuif, The Enhancer of split transcription factor Her8a is a novel dimerisation partner for Her3 that controls anterior hindbrain neurogenesis in zebrafish. *BMC Dev. Biol.* **11**, 27 (2011).

50. S.-F. Tzeng, Inhibitors of DNA binding in neural cell proliferation and differentiation. *Neurochem. Res.* **28**, 45–52 (2003).

51. G. Bai, N. Sheng, Z. Xie, W. Bian, Y. Yokota, R. Benezra, R. Kageyama, F. Guillemot, N. Jing, Id sustains Hes1 expression to inhibit precocious neurogenesis by releasing negative autoregulation of Hes1. *Dev. Cell* **13**, 283–297 (2007).

52. M. Boareto, D. Iber, V. Taylor, Differential interactions between Notch and ID factors control neurogenesis by modulating Hes factor autoregulation. *Development* **144**, 3465–3474 (2017).

53. U. Coppola, J. S. Waxman, Origin and evolutionary landscape of Nr2f transcription factors across Metazoa. *PLOS ONE* **16**, e0254282 (2021).

54. L. S. Tang, H. M. Alger, F. A. Pereira, COUP-TFI controls Notch regulation of hair cell and support cell differentiation. *Development* **133**, 3683–3693 (2006).

55. C. Montemayor, O. A. Montemayor, A. Ridgeway, F. Lin, D. A. Wheeler, S. D. Pletcher, F. A. Pereira, Genome-wide analysis of binding sites and direct target genes of the orphan nuclear receptor NR2F1/COUP-TFI. *PLOS ONE* **5**, e8910 (2010).

56. A. Hollnagel, V. Oehlmann, J. Heymer, U. Rüther, A. Nordheim, Id genes are direct targets of bone morphogenetic protein induction in embryonic stem cells. *J. Biol. Chem.* **274**, 19838–19845 (1999).

57. G. Zhang, M. Ferg, L. Lübke, M. Takamiya, T. Beil, V. Gourain, N. Diotel, U. Strähle, S. Rastegar, Bone morphogenetic protein signaling regulates Id1-mediated neural stem cell quiescence in the adult zebrafish brain via a phylogenetically conserved enhancer module. *Stem Cells* **38**, 875–889 (2020).

58. G. Zhang, L. Lübke, F. Chen, T. Beil, M. Takamiya, N. Diotel, U. Strähle, S. Rastegar, Neuron-radial glial cell communication via BMP/Id1 signaling is key to long-term maintenance of the regenerative capacity of the adult zebrafish telencephalon. *Cells* **10**, 2794 (2021).

59. R.-F. Li, T.-Y. Wu, Y.-Z. Mou, Y.-S. Wang, C.-L. Chen, C.-Y. Wu, Nr2f1b control venous specification and angiogenic patterning during zebrafish vascular development. *J. Biomed. Sci.* **22**, 104 (2015).

60. G. Chowdhury, K. Umeda, T. Ohyanagi, K. Nasu, K. Yamasu, Involvement of nr2f genes in brain regionalization and eye development during early zebrafish development. *Dev. Growth Differ.* **66**, 145–160 (2024).

61. S. Katz, D. Cussigh, N. Urbán, I. Blomfield, F. Guillemot, L. Bally-Cuif, M. Coolen, A nuclear role for miR-9 and argonaute proteins in balancing quiescent and activated neural stem cell states. *Cell Rep.* **17**, 1383–1398 (2016).

62. H. Shibata, Z. Nawaz, S. Y. Tsai, B. W. O'Malley, M.-J. Tsai, Gene silencing by chicken ovalbumin upstream promoter-transcription factor i (COUP-TFI) is mediated by transcriptional corepressors, nuclear receptor-corepressor (N-CoR) and silencing mediator for retinoic acid receptor and thyroid hormone receptor (SMRT). *Mol. Endocrinol.* **11**, 714–724 (1997).

63. C. E. Muller-Sieburg, H. B. Sieburg, J. M. Bernitz, G. Cattarossi, Stem cell heterogeneity: Implications for aging and regenerative medicine. *Blood* **119**, 3900–3907 (2012).

64. I. Imayoshi, M. Sakamoto, M. Yamaguchi, K. Mori, R. Kageyama, Essential roles of Notch signaling in maintenance of neural stem cells in developing and adult brains. *J. Neurosci.* **30**, 3489–3498 (2010).

65. O. Ehm, C. Göritz, M. Covic, I. Schäffner, T. J. Schwarz, E. Karaca, B. Kempkes, E. Kremmer, F. W. Pfrieger, L. Espinosa, A. Bigas, C. Giachino, V. Taylor, J. Frisén, D. C. Lie, RBPJκ-dependent signaling is essential for long-term maintenance of neural stem cells in the adult hippocampus. *J. Neurosci.* **30**, 13794–13807 (2010).

66. J. L. Ables, N. A. Decarolis, M. A. Johnson, P. D. Rivera, Z. Gao, D. C. Cooper, F. Radtke, J. Hsieh, A. J. Eisch, Notch1 is required for maintenance of the reservoir of adult hippocampal stem cells. *J. Neurosci.* **30**, 10484–10492 (2010).

67. T. E. Anthony, H. A. Mason, T. Gridley, G. Fishell, N. Heintz, Brain lipid-binding protein is a direct target of Notch signaling in radial glial cells. *Genes Dev.* **19**, 1028–1033 (2005).

68. M. Namihira, J. Kohyama, K. Semi, T. Sanosaka, B. Deneen, T. Taga, K. Nakashima, Committed neuronal precursors confer astrocytic potential on residual neural precursor cells. *Dev. Cell* **16**, 245–255 (2009).

69. L. Dang, K. Yoon, M. Wang, N. Gaiano, Notch3 signaling promotes radial glial/progenitor character in the mammalian telencephalon. *Dev. Neurosci.* **28**, 58–69 (2006).

70. U. Jadhav, M. Saxena, N. K. O'Neill, A. Saadatpour, G.-C. Yuan, Z. Herbert, K. Murata, R. A. Shivdasani, Dynamic reorganization of chromatin accessibility signatures during dedifferentiation of secretory precursors into $Lgr5^+$ intestinal stem cells. *Cell Stem Cell* **21**, 65–77.e5 (2017).

71. S. Bonzano, I. Crisci, A. Podlesny-Drabiniok, C. Rolando, W. Krezel, M. Studer, S. De Marchis, Neuron-astroglia cell fate decision in the adult mouse hippocampal neurogenic Niche is cell-intrinsically controlled by COUP-TFI in vivo. *Cell Rep.* **24**, 329–341 (2018).

72. V. Silva-Vargas, A. R. Maldonado-Soto, D. Mizrak, P. Codega, F. Doetsch, Age-dependent niche signals from the choroid plexus regulate adult neural stem cells. *Cell Stem Cell* **19**, 643–652 (2016).

73. J. Zhang, S. Fukuhara, K. Sako, T. Takenouchi, H. Kitani, T. Kume, G. Y. Koh, N. Mochizuki, Angiopoietin-1/Tie2 signal augments basal Notch signal controlling vascular quiescence by inducing delta-like 4 expression through AKT-mediated activation of β-catenin. *J. Biol. Chem.* **286**, 8055–8066 (2011).

74. D. R. M. Seib, N. S. Corsini, K. Ellwanger, C. Plaas, A. Mateos, C. Pitzer, C. Niehrs, T. Celikel, A. Martin-Villalba, Loss of Dickkopf-1 restores neurogenesis in old age and counteracts cognitive decline. *Cell Stem Cell* **12**, 204–214 (2013).

75. L. Xu, Y. Chen, Y. Huang, E. Sandanaraj, J. S. Yu, R. Y.-T. Lin, P. Dakle, X.-Y. Ke, Y. K. Chong, L. Koh, A. Mayakonda, K. Nacro, J. Hill, M.-L. Huang, S. Gery, S. W. Lim, Z. Huang, Y. Xu, J. Chen, L. Bai, S. Wang, H. Wakimoto, T. T. Yeo, B. T. Ang, M. Müschen, C. Tang, T. Z. Tan, H. P. Koeffler, Topography of transcriptionally active chromatin in glioblastoma. *Sci. Adv.* **7**, eabd4676 (2021).

76. M. S. Sosa, F. Parikh, A. G. Maia, Y. Estrada, A. Bosch, P. Bragado, E. Ekpin, A. George, Y. Zheng, H.-M. Lam, C. Morrissey, C.-Y. Chung, E. F. Farias, E. Bernstein, J. A. Aguirre-Ghiso, NR2F1 controls tumour cell dormancy via SOX9- and RARβ-driven quiescence programmes. *Nat. Commun.* **6**, 6170 (2015).

77. S.-Y. Yeo, M. Kim, H.-S. Kim, T.-L. Huh, A. B. Chitnis, Fluorescent protein expression driven by her4 regulatory elements reveals the spatiotemporal pattern of Notch signaling in the nervous system of zebrafish embryos. *Dev. Biol.* **301**, 555–567 (2007).

78. C. S. McGinnis, L. M. Murrow, Z. J. Gartner, DoubletFinder: Doublet detection in single-cell RNA sequencing data using artificial nearest neighbors. *Cell Syst.* **8**, 329–337.e4 (2019).

79. J. D. Welch, V. Kozareva, A. Ferreira, C. Vanderburg, C. Martin, E. Z. Macosko, Single-cell multi-omic integration compares and contrasts features of brain cell identity. *Cell* **177**, 1873–1887.e17 (2019).

80. H. T. N. Tran, K. S. Ang, M. Chevrier, X. Zhang, N. Y. S. Lee, M. Goh, J. Chen, A benchmark of batch-effect correction methods for single-cell RNA sequencing data. *Genome Biol.* **21**, 12 (2020).

81. S. Aibar, C. B. González-Blas, T. Moerman, V. A. Huynh-Thu, H. Imrichova, G. Hulselmans, F. Rambow, J.-C. Marine, P. Geurts, J. Aerts, J. van den Oord, Z. K. Atak, J. Wouters, S. Aerts, SCENIC: Single-cell regulatory network inference and clustering. *Nat. Methods* **14**, 1083–1086 (2017).

82. G. A. Rousselet, C. R. Pernet, R. R. Wilcox, Beyond differences in means: robust graphical methods to compare two groups in neuroscience. *Eur. J. Neurosci.* **46**, 1738–1748 (2017).
